# Exposure to Metals, Pesticides, and Air Pollutants: Focus on Resulting DNA Methylation Changes in Neurodegenerative Diseases

**DOI:** 10.3390/biom14111366

**Published:** 2024-10-27

**Authors:** Andrea Stoccoro, Fabio Coppedè

**Affiliations:** 1 Laboratory of Medical Genetics, Department of Translational Research and of New Surgical and Medical Technologies, Medical School, University of Pisa, Via Roma 55, 56126 Pisa, Italy; andrea.stoccoro@unipi.it; 2 Interdepartmental Research Center of Biology and Pathology of Aging, University of Pisa, 56126 Pisa, Italy

**Keywords:** metals, pesticides, air pollutants, particulate matter, neurodegenerative diseases, epigenetics, DNA methylation, Alzheimer’s disease, Parkinson’s disease, amyotrophic lateral sclerosis

## Abstract

Individuals affected by neurodegenerative diseases, including Alzheimer’s disease (AD), Parkinson’s disease (PD), and amyotrophic lateral sclerosis (ALS), are dramatically increasing worldwide. Thus, several efforts are being made to develop strategies for stopping or slowing the spread of these illnesses. Although causative genetic variants linked to the onset of these diseases are known, they can explain only a small portion of cases. The etiopathology underlying the neurodegenerative process in most of the patients is likely due to the interplay between predisposing genetic variants and environmental factors. Epigenetic mechanisms, including DNA methylation, are central candidates in translating the effects of environmental factors in genome modulation, and they play a critical role in the etiology of AD, PD, and ALS. Among the main environmental exposures that have been linked to an increased risk for these diseases, accumulating evidence points to the role of heavy metals, pesticides, and air pollutants. These compounds could trigger neurodegeneration through different mechanisms, mainly neuroinflammation and the induction of oxidative stress. However, increasing evidence suggests that they are also capable of inducing epigenetic alterations in neurons. In this article, we review the available literature linking exposure to metals, pesticides, and air pollutants to DNA methylation changes relevant to neurodegeneration.

## 1. Introduction

The increased number of age-related diseases is having an ever-increasing impact on public healthcare systems and social wellness around the world. This is mainly due to improvements in the medical field that allow people to live longer. In particular, it is estimated that about 50 million people worldwide are suffering from Alzheimer’s disease (AD) or other forms of dementia, and it is estimated that this number could triple by 2050 [1]. The prevalence of Parkinson’s disease (PD) has doubled since the early 1990s and is expected to double again by 2040 [2]. Similarly, deaths due to motor neuron diseases, including amyotrophic lateral sclerosis (ALS), are estimated to have increased by 12.4% from 1990 to 2019 [3]. So, the need to find cures for neurodegenerative diseases, or to design strategies to prevent or at least delay their onset, is becoming increasingly pressing. For this reason, numerous efforts are being made to find ways to cope with this. The etiology of neurodegenerative diseases is still poorly understood, but the view that a pivotal role is played by the interplay between predisposing genetic variants and environmental factors is well accepted [4]. Several environmental factors have been suggested to play a role in this interplay, including lifestyles, diet, physical activity, and trauma, and a critical role seems to be played by adverse environmental exposure. In this way, the 2020 Lancet Commission on Dementia Prevention updated the modifiable risk factors for dementia proposed in 2017, adding air pollution as one of the three new risk factors due to strong emerging data supporting its association with AD risk [5]. In addition, various metals and pesticides have long been considered risk factors for neurodegenerative diseases [6]. A better understanding of the etiopathology of non-genetic modifiable risk factors for age-related neurodegenerative diseases could greatly contribute to reducing the risk of developing such disorders. Within this context, increasing evidence points to epigenetic consequences following exposure to metals, pesticides, and air pollutants, i.e., the ability of these compounds to induce epigenetic modifications ultimately resulting in changes in gene expression. In the present article, we will focus on the DNA methylation changes induced by exposure to metals, pesticides, and air pollutants in the context of major neurodegenerative diseases, namely AD, PD, and ALS.

## 2. Metals, Pesticides, and Air Pollution as Risk Factors for Age-Related Neurodegenerative Diseases

Neurodegenerative diseases, including AD, PD, ALS, and others, are a group of disorders of the central nervous system characterized by progressive neuronal loss; they are incurable and often fatal within a few years after diagnosis. Although these diseases are characterized by specific pathogenetic alterations, they share some basic pathological mechanisms, including the intraneuronal and/or extracellular accumulation of proteins which determines an altered viability of neurons [7]. Indeed, AD is characterized by the accumulation of extracellular amyloid plaques formed by β-amyloid peptide (Aβ) deposits and by the aggregation of hyperphosphorylated tau protein to form neurofibrillary tangles within neurons. PD is instead characterized by the accumulation of intraneuronal α-synuclein proteins in the substantia nigra, leading to the loss of dopaminergic neurons. Protein aggregation is a hallmark of ALS too, as amyloid aggregates from different proteins, such as TDP-43, C9ORF72 dipeptide repeats, and FUS, are always reported in specimens from ALS patients [8].

Monogenic forms of AD, PD, and ALS account for only a minority of the cases, and in most patients, the disease results from complex gene–gene and gene–environment interactions superimposed on age-related neuronal dysfunction. Indeed, only about 1% of AD results from autosomal dominant mutations in one of three causative genes, namely *APP*, *PSEN1*, and *PSEN2*, coding for the amyloid precursor protein and for presenilin proteins, respectively, all involved in the amyloid cascade that leads to increased production and deposition of the Aβ peptide [9]. Numerous genes have been linked to monogenic forms of PD, including autosomal dominant mutations in *SNCA*, *LRRK2*, *CHCHD2*, and *VPS35*, recessive mutations in *PARKIN*, *DJ1*, and *PINK1*, and mutations in other genes linked to atypical parkinsonism. Collectively, monogenic forms of PD account for almost 15% of the cases [10]. Similarly, familial forms account for almost 10% of ALS cases. They often result from highly penetrant mutations in *SOD1*, *FUS*, *TARDBP*, and *C9orf72* genes. Rare or private variants in numerous additional genes explain the rest of monogenic ALS [11].

Apart from monogenic forms, most AD, PD, and ALS cases are sporadic. Large-scale genome-wide association studies (GWASs) and exome sequencing approaches are increasingly revealing both rare and common variants in numerous genes that are linked to these conditions. Indeed, oligogenic and polygenic inheritance models and complex gene–environment interactions have been proposed to explain the majority of AD, PD, and ALS cases. Metals, pesticides, and air pollution are among the environmental factors that have been largely proposed as risk factors for neuronal impairment and neurodegeneration [10,12,13].

Indeed, the central nervous system seems to be particularly sensitive to reactive oxygen species (ROS) and neuroinflammation induced by metals, pesticides, and air pollutants [14]. Neuroinflammation and increased ROS production are likely due to the enhanced activation of glial cells, which are involved in the innate immune response in the central nervous system. Increased activation of glial cells leads to the elevated production of chemokines and several proinflammatory cytokines, including TNFα, IL-6, IL-1β, cyclooxygenase-2, and inducible nitric oxide synthase, ultimately leading to neuroinflammation [15,16]. In vivo studies have demonstrated increased cytokine and chemokine production across various brain regions, such as the hippocampus, striatum, and cortex, which are brain regions closely associated with neurodegenerative diseases [17,18]. It has also been reported that neuroinflammation can induce alterations in the blood–brain barrier through the production of antibodies against tight junctions of the endothelial cells, potentially contributing to the formation of neuropathological hallmarks of AD and PD in children residing in Metropolitan Mexico City (MMC), an area with high concentrations of air pollution and metals [19]. Metals, pesticides, and air pollution could also induce protein aggregation and DNA damage, which are molecular alterations frequently identified in the brains of individuals with neurodegenerative diseases. For example, post-mortem brain samples of young adults residing in MMC showed the presence of hyperphosphorylated tau proteins and Aβ peptides along with alterations in chromatin state and increased DNA damage when compared to residents in areas with low pollution [20]. Another suggested molecular mechanism underlying environment-induced neurodegeneration is mitochondrial dysfunction. Indeed, mitochondria are primary targets of oxidative stress resulting from exposure to adverse environmental factors [21]. Due to the high energy demand of the brain, mitochondrial dysfunction and the subsequent reduction in ATP levels can markedly impair brain function, promoting neurodegenerative processes [22]. There is also evidence that exposure during gestation could have adverse neurotoxic effects in utero leading to a wide variety of neurodevelopmental disorders, including autism spectrum disorders or developmental delays [23]. In line with this, the Developmental Origins of Health and Disease (DOHaD) theory, proposed by David Barker in the early 2000s, hypothesizes that exposure to environmental risk factors during embryo development and in the first years of infancy can increase the risk of developing chronic diseases in adulthood. In this regard, animal studies showed that in utero exposure to various environmental pollutants, including metals and pesticides, induced impairment in learning and memory, as well as the formation of biological markers of AD and PD, such as the accumulation of amyloid plaques and dopaminergic impairments [24].

Several metals have been involved in neurodegenerative processes. Among them, increasing evidence points to the role of lead (Pb) in inducing the neurodegenerative process, mainly associated with PD and ALS [25]. Another metal suspected to contribute to neurodegeneration, particularly PD and ALS, is mercury (Hg) [26,27,28,29]. Arsenic (As) too is a metal known to cause various neurological disorders mainly through the induction of cellular toxicity, hyperproduction of ROS, and inflammation, and studies in animal models and in human subjects showed that exposure to As can increase the risk of developing AD [30,31,32]. Other metals that have been suggested to increase the risk of neurodegenerative diseases are nickel for AD and PD [33], chromium for AD [34], copper (Cu) for AD and PD [35], manganese (Mn) for PD and ALS [36,37], aluminum for AD [38], and iron for AD, PD, and ALS [39]. Overall, recent systematic reviews and meta-analyses revealed that owing to the large heterogeneity among study designs, methods for assessing exposure, routes of exposure, and other methodological issues, the available literature is often insufficient to establish a clear association between a specific metal exposure and the risk of a particular neurodegenerative disease, although potential associations such as that between lead exposure and risk of both PD and ALS are emerging. Therefore, further studies are warranted to clarify this issue, including studies investigating the capabilities of metals and metalloids to induce disease-related epigenetic changes [12,40,41].

Pesticides are well-known neurotoxicants that have been frequently associated with neurodegenerative diseases, particularly PD [42]. Indeed, several pesticides, such as 1-methyl-4-phenyl-1,2,3,6-tetrahydropyridine (MPTP), paraquat, maneb, organochlorines, and rotenone, seem to be particularly harmful to dopaminergic neurons, causing a great impairment in the neuronal viability of the substantia nigra [43]. Pesticides have also been found to trigger the deposition of amyloid plaques and neurofibrillary tangles, thus increasing the risk for AD [44]. Moreover, a meta-analysis of studies that estimated the likelihood of exposure of individuals to various pollutants suggested that pesticide exposure is linked to an increased risk of developing ALS [45]. Further evidence of a potential association comes from a recent systematic review and meta-analysis showing that pesticide exposure can represent a risk factor for both ALS onset and progression [41].

The potential role of air pollutants in neurodegenerative diseases is suggested by epidemiological studies that found an increased incidence of AD in individuals living in areas with high levels of air pollution [46]. Air pollution can contain various chemicals, including particulate matter (PM), such as PM_10_ and PM_2.5_, and gaseous substances such as O_3_, SO_2_, and NO_2_, which could trigger the neurodegenerative process by inducing inflammation and oxidative stress [47]. Direct evidence of the role of air pollutants in the neurodegenerative process has been obtained from animal and post-mortem human studies showing that exposure to PM of different sizes increased protein aggregation, such as α-synuclein and β-amyloid peptides, potentially contributing to the etiopathogenesis of AD and PD [48]. More recent meta-analyses revealed a significant positive association between long-term PM_2.5_ exposure and all-cause dementia, AD, and PD [49,50]. Moreover, exposure to various air pollutants, including PM_2.5_ and NO_2_, has been related to increased risk of ALS [51] and to ALS mortality [52]. Recently, neuropathological hallmarks of AD, PD, and ALS, including peptide beta amyloid, hyperphosphorylated tau, α-synuclein, and TAR DNA-binding protein-43 (TDP-43), were identified in the post-mortem brain samples of children and young adults residing in highly polluted areas [53]. They were all residents of MMC, an area with high levels of air pollutants, including PM_2.5_, combustion and friction ultrafine PM, and industrial nanoparticles (NPs). Particularly, authors identified neuropathological hallmarks associated with the presence of NPs along with various metals in brain tissues [53]. Moreover, increased levels of TDP-43 were found in the cerebrospinal fluid of young residents in MMC when compared to residents in areas with low pollution [54]. The results of these studies demonstrate the profound impact of air pollution exposure on children’s central nervous systems, emphasizing how early exposure increases the risk of developing neurodegenerative diseases later in life. Of note, in 2020, the Lancet commission on dementia prevention, intervention, and care included air pollution as a novel risk factor for dementia [5]. Therefore, it is essential to implement public health measures as soon as possible to prevent or at least limit as much as possible, already during childhood, the exposure to environmental pollutants that increase the risk of developing neurodegenerative diseases.

## 3. DNA Methylation

**Figure 1:**
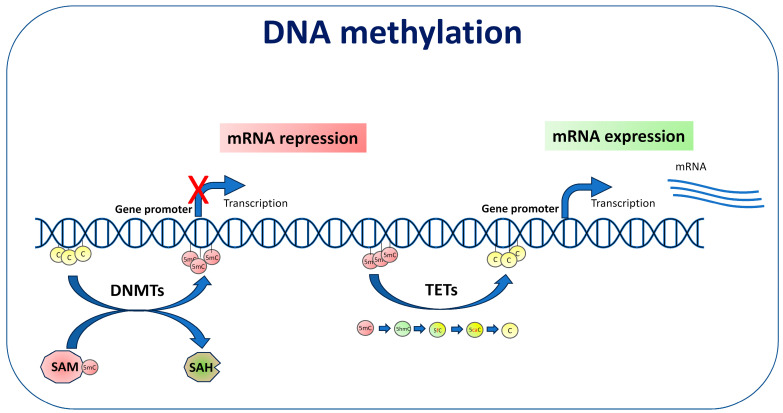
DNA methylation is mediated by DNA methyltransferases (DNMTs) which obtain the methyl group from S-adenosylmethionine (SAM) which is then converted into S-adenosylhomocysteine (SAH). Methylation of CpG sites within the promoter region is usually associated with repression of gene expression. Active DNA demethylation is catalyzed by ten-eleven translocation (TET) proteins. TETs mediate the formation of 5-hydroxymethylcytosine (5hmC) which can be further converted to 5-formylcytosine (5fC) and then to 5-carboxycytosine (5caC). The latter can in turn be removed by the DNA base excision repair system and replaced by cytosine in the base sequence. The presence of hypomethylated DNA is usually associated with transcriptional activation.

DNA methylation is an epigenetic mechanism that consists in the addition of a methyl group to the fifth carbon of a cytosine that is followed by a guanine residue, the CpG site, leading to the formation of 5-methylcytosine (5mC). There are regions in the genome rich in CpG sites, the so-called CpG islands, that are mainly located in the promoter sequence of the genes. Usually, when the CpG islands are methylated, the expression of the associated genes is repressed, while demethylated CpG islands, by allowing the binding of transcription factors to the DNA, are usually associated with active gene transcription [55]. The methylation of a cytosine not followed by a guanine in the DNA sequence is called non-CpG methylation. Non-CpG DNA methylation occurs preferentially in certain cell types, including pluripotent stem cells, glial cells, and neurons. In neurons, both CpG and non-CpG methylation are responsible for gene expression levels and neural plasticity [56]. 5-hydroxymethylcytosine (5hmC) is another modification that can occur on cytosine. 5hmC was originally considered as an intermediate product of 5mC demethylation, but it is nowadays considered as a distinct epigenetic mark mainly associated with active transcriptional DNA [57]. The formation of 5mC is mediated by a group of enzymes called DNA methyltransferases (DNMTs) which obtain methyl groups from *S*-adenosylmethionine (SAM), a compound synthesized in the one-carbon metabolism that is strictly dependent on folate intake. Three main DNMTs are involved in DNA methylation, namely DNMT1, involved in the maintenance of DNA methylation, and DNMT3A and DNMT3B, which are responsible for de novo DNA methylation. Another class of enzymes, called ten-eleven translocation (TET) proteins, is involved in the active demethylation process. TETs mediate the formation of 5hmC, which can be further catalyzed to 5-formylcytosine (5fC) and then to 5-carboxycytosine (5caC), which in turn can be removed by base excision repair and replaced by cytosine in the base sequence (Figure 1).

In recent years, it has emerged that epigenetic mechanisms could also act inside mitochondria, potentially regulating the expression levels of genes encoded by the mitochondrial DNA (mtDNA) and/or protecting the mtDNA from oxidative DNA damage [58,59]. Mitochondrial epigenetics, also named “mitoepigenetics”, focuses mainly on DNA methylation and non-coding RNA activities, as mtDNA lacks histones. DNMT1, DNMT3A, DNMT3B, and TET enzymes have been found inside mitochondria, and methylation of the mtDNA non-coding control region, the D-loop sequence that has a similar function to the promoter regions of nuclear genes, has been associated with both mtDNA gene expression and mtDNA replication [58]. Indeed, impaired methylation of mtDNA regions, and in particular of the D-loop region, has been observed in several human pathologies, including neurodegenerative disorders [58]. Moreover, “mitoepigenetics” is also considered the crosstalk between the nucleus and mitochondria from an epigenetic point of view. Indeed, mitochondria synthesize several molecules for epigenetic reactions, including ATP and acetyl-coA, and in the nucleus, the proteins necessary for mtDNA transcription and replication are synthesized [60].

The role of epigenetics in physiological and pathological conditions has had a great impact on the scientific community thanks to the advent of new technologies for the studies of such mechanisms. Regarding DNA methylation, alongside global methylation analysis that allows the estimation of the overall content of 5mC of DNA without giving information on methylation levels of specific loci, studies can be focused on DNA methylation of specific genes, through the so-called candidate gene approach analyses. With the advent of novel genomic technologies in the last few years, specific loci throughout the genome can be analyzed in a single analysis using genome-wide DNA methylation approaches. The latter permits epigenome-wide association (EWAS) investigations, i.e., the analysis of DNA methylation at the genome-wide level in different individuals to derive associations between epigenetic variation and a particular identifiable phenotype, to be performed [61].

## 4. DNA Methylation and Neurodegeneration

Epigenetic mechanisms, and particularly DNA methylation, play a pivotal role in neural function, neuronal plasticity, and memory formation [62]. Moreover, age, the most known risk factor for neurodegenerative diseases, is characterized by the accumulation of epigenetic modifications that are associated with a decline in cognitive capacities [63]. In this regard, it has been reported that patients with neurodegenerative diseases, including AD, PD, and ALS, are characterized by an accelerated epigenetic age, evaluable through the analysis of several CpG sites throughout the genome, compared to cognitive control subjects [64]. So, it is not surprising that epigenetic mechanisms have been proposed as pivotal players in the etiopathogenesis of neurodegenerative diseases.

Early experiments aimed at identifying epigenetic alterations in AD were focused on the evaluation of DNA methylation in genes involved in the pathway of amyloid peptide formation. The first study in this field identified lower DNA methylation levels of the *APP* gene in the brain of an AD patient when compared to a neurologically healthy subject [65]. Then, in vitro and in vivo studies showed that exposure to environmental factors able to influence epigenetic mechanisms, including deficiency of B-group vitamins or early exposure to lead, induced altered methylation levels in genes involved in the Aβ-peptide synthesis, including *PSEN1*, *BACE1*, and *APP* [66,67]. With the advent of techniques that allowed the investigation at the genome-wide level, several loci have been epigenetically associated not only with AD status, i.e., their capacity to discriminate between cases and control subjects, but also with the progression of the disease [68]. EWAS studies performed in post-mortem AD brains revealed hundreds of differentially methylated genes and CpG sites with respect to control brains [69], so researchers are increasingly investigating DNA methylation changes in peripheral blood samples of AD patients to identify early peripheral biomarkers of the disease [70]. Although an epigenetic signature for AD has not yet been identified, there is a consensus that epigenetic mechanisms are involved in AD pathology and that they could mediate the effects of adverse environmental exposure [12].

The first studies aimed at identifying epigenetic alterations in PD searched for DNA methylation patterns of the *SNCA* gene in specimens from PD and healthy control subjects [71]. Many investigators identified hypomethylation of the *SNCA* gene in both brain and peripheral blood cells of PD individuals, albeit with some conflicting results [72]. As for AD, subsequent EWAS investigations in PD brains revealed several genes and CpG sites potentially associated with the disease [73,74]. Several DNA methylation changes were also observed in blood samples of PD patients [75].

Several studies conducted in both peripheral and central nervous system tissues have suggested that altered methylation levels could contribute also to the etiopathogenesis of ALS [76,77,78]. In particular, a global increase in DNA methylation levels has been observed in both sporadic and familial forms of the disease [79,80,81], and recent large-scale EWAS and gene expression studies revealed that most of the observed methylation changes involve genes important for immune, inflammatory, and metabolic functions [82].

Considering the fundamental role of mitochondria in aging and neuronal biology, the role of mitoepigenetics is increasingly gaining interest in the field of neurodegenerative diseases. Alterations in mitochondrial DNA methylation levels have been associated with senescence and aging, two processes highly linked to neurodegenerative diseases [83,84,85]. Moreover, altered levels of mitochondrial DNA methylation in in vitro and in vivo models, as well as in patient tissues, of AD, PD, and ALS have been reported. The first piece of evidence suggesting a role for mtDNA methylation in neurodegenerative diseases was obtained in a study involving mouse models of ALS and human patients [86]. The authors observed increased levels of mtDNA methylation that were associated with the apoptotic process of motor neurons in ALS mice when compared to control mice. Similar levels of mtDNA methylation were found in human cortical motor neurons of ALS patients [86]. Furthermore, altered levels of the D-loop region and the mitochondrial gene *MT-RNR2* were identified in muscle tissues and spinal cord of transgenic mice carrying mutations in the *SOD1* gene [87]. Altered levels of D-loop methylation were also found in the peripheral blood of individuals with *SOD1* mutations, including both ALS patients and asymptomatic subjects, compared to family members without the mutations and to ALS patients with mutations in the *FUS*, *TARDBP*, and *C9orf72* genes [88]. Altered levels of D-loop methylation were also observed in patients with sporadic ALS [89]. More recently, an inverse correlation between D-loop methylation levels and disease duration in ALS patients with mutations in *SOD1* and *C9orf72* was detected [90]. Altered mtDNA methylation levels have also been suggested for AD and, to a lesser extent, for PD. Methylation levels of mtDNA were found to change with disease progression in the entorhinal cortex of patients with AD-related pathology, as well as in brain samples of AD mice [91,92]. Moreover, we observed that peripheral blood D-loop methylation levels changed in individuals at different stages of the AD pathology [93,94]. Decreased methylation levels of the D-loop region were found in the substantia nigra of PD patients compared to controls [91]. Furthermore, global decreased mtDNA methylation was detected in peripheral blood and induced pluripotent stem cell (iPSC)-derived midbrain neurons from PD patients when compared to controls [95]. However, other studies did not reveal mtDNA methylation alterations in the prefrontal cortex [96] or peripheral blood of PD patients when compared to control subjects [97,98].

## 5. DNA Methylation Changes Induced by Metals, Pesticides, and Air Pollutants in Neurodegenerative Diseases

The mechanisms underlying the adverse effects of environmental pollutants on brain cells, and particularly on the neurodegenerative process, remain poorly understood, although the majority of data collected until now suggest that a main role is played by inflammation and oxidative stress [6]. However, several pieces of evidence point to a potential role of environmental exposure in inducing age-related neurodegenerative disease through DNA methylation alterations. Although there are obvious challenges in identifying clear causal associations between exposure and age-related disease through the induction of aberrant epigenetic mechanisms, some researchers tried to shed light on this issue through in vitro and in vivo investigations as well as performing studies in human specimens from neurodegenerative disease patients.

### 5.1. In Vitro and In Vivo Studies

A seminal paper that identified a link between lead-induced epigenetic modifications and AD was published in 2008. In that study, Wu and collaborators showed that in aged (23-year-old) monkeys exposed to Pb as infants, the expression levels of AD-related genes, including *APP* and *BACE1*, as well as their transcriptional regulator (Sp1), were elevated [67]. At the same time, DNMT1 activity was decreased, suggesting that Pb-linked altered gene and protein expression levels could be mediated by epigenetic mechanisms. In the same animals, authors identified altered expression of other genes and proteins involved in epigenetic regulation, including *Dnmts*, the methyl-CpG binding domain protein-encoding gene *MeCP2*, and proteins involved in histone modifications [99]. Moreover, treatment of neuroblastoma cells with Mn revealed altered methylation levels in genes relevant to PD pathogenesis, including *PINK1* and *PARK2* [100]. Furthermore, Yang and collaborators identified altered DNA methylation levels at several loci in the substantia nigra of mice exposed to manganese [101]. In a following study, the effects of manganese and of 1-methyl-4-phenylpyridinium (MPP^+^), which was used as an herbicide under the trade name of cyperquat (an analog of paraquat), treatments were investigated in dopaminergic neurons, and the results were confirmed in a mouse model of PD [102]. Mn and MPP^+^ induced distinct morphological and electrophysiological alterations in dopaminergic neurons, as well as distinct transcriptomic signatures. Moreover, genome-wide DNA methylation analysis showed altered methylation levels following MPP^+^ and Mn treatments, both specific and in common, in genes involved in different pathways, including neuronal activity, mitochondrial function, and DNA repair [102]. Furthermore, mtDNA methylation patterns have been observed as a target of iron-induced neurodegeneration. Indeed, it has been observed that rats receiving iron in the neonatal period at doses that induced neurodegenerative processes in adulthood had altered global mtDNA methylation and hydroxymethylation, potentially impairing mitochondria metabolism [103].

Dieldrin is another pesticide widely used from the 1950s to early 1970s that is now banned from most countries around the world and has been strictly associated with an increased risk of developing PD [104]. It has been proposed that developmental exposure to dieldrin could induce PD through DNA methylation alterations [105]. Indeed, the ventral mesencephalon of pup mice developmentally exposed to dieldrin exhibited several differentially methylated genes, including the *Nr4a2* and *Lmx1b* genes, which are involved in dopaminergic neuron development and maintenance [105]. Interestingly, polymorphisms and reduced expression levels of the *NR4A2* gene have been associated with an increased risk of developing PD [106], and decreased *LMX1B* gene expression levels were found in post-mortem brains of PD patients [107]. More recently, the same research group identified sex-specific dieldrin-induced DNA methylation changes in pup mice exposed to the pesticide in utero and after birth until 36 weeks of age [108]. Particularly, authors observed dieldrin-induced DNA methylation changes at different time points, at birth, 6 weeks, 12 weeks, and 36 weeks of age, in loci associated with pathways related to neurodevelopment, dopaminergic neuron differentiation, synaptogenesis, and synaptic plasticity [108]. It has been also observed that another PD-linked pesticide, rotenone, can induce altered methylation in specific genomic regions involved in neuronal function and PD pathogenesis, including *HCN2* and *NEFM*, and their expression was increased in brain regions associated with PD [109]. Of note, *HCN2* belongs to a family of genes encoding for a hyperpolarization-activated cation channel, whose expression in the basal ganglia nuclei decreases during PD onset and development, leading to motor and non-motor symptoms of the disease [110]. Moreover, previous reports associated *NEFM* gene variants with PD [111] and identified altered *NEFM* gene expression levels in the substantia nigra of PD patients [112]. Rotenone was also able to alter DNA methylation in the proximity of CTCF binding sites, with CTCF being a regulatory protein that binds DNA to control spatial organization and transcription, in genes associated with PD, including *PARK2* [113]. Furthermore, fenpropathrin, a volatile pyrethroid insecticide, seems to increase the risk of PD through epigenetic modifications. Indeed, in the midbrain of mice exposed to fenpropathrin, increased methylation and decreased expression of the *Ambra1* gene, which is involved in the mitophagy of dopaminergic neurons, were detected [114]. Interestingly, the administration of a demethylating agent was able to upregulate *Ambra1* expression, thus reducing mitophagy and protecting dopaminergic neurons against fenpropathrin-induced damage. This is a notable finding, especially given that *Ambra1* is known to play a key role in the PD pathological processes [115].

Although several pieces of evidence link exposures to air pollutants and an increased risk of neurodegenerative diseases, only few studies have been performed until now searching for DNA methylation alteration induced by such exposure in neurodegeneration. Altered methylation levels of two genes known to be linked to AD, *ABCA7* and *PYK2*, have been found in hippocampal samples of mice exposed to traffic-related air pollution, which developed impaired memory function and enhanced neuroinflammation and oxidative stress responses [116].

Investigations in cell cultures and animal models of neurodegenerative diseases have been performed to investigate the potential contribution of metal, pesticide, and air pollutant exposure to the neurodegenerative process through DNA methylation alterations (Table 1). Overall, the experimental models employed were very different among the twelve studies collected. Indeed, eight studies were performed in animal models, two in brain tissues from monkeys, one in hippocampal specimens of rats, three in the substantia nigra of mice, one in the midbrain of mice, and one in hippocampal samples of mice. Four studies were performed in cell cultures as models of PD, including one in human neuroblastoma cells, one in rat dopaminergic cells, and two in human embryonic kidney cells. Thus, these studies provide information on the exposure that induces specific alterations of neurodegenerative diseases and therefore are not representative of the entire phenotypic spectrum that characterizes AD, PD, and ALS. So, it is difficult to understand the real extent to which DNA methylation could contribute to the etiology of neurodegenerative diseases following exposure to metals, pesticides, and air pollution from in vitro and in vivo studies. These studies suggest the existence of correlations among adverse exposure, DNA methylation changes, and neuropathology but cannot provide the causality of such interactions. However, they can suggest epigenetic targets involved in at least some neuropathological pathways underlying the diseases that can be used in translational investigations in human studies.

### 5.2. Human Studies

In addition to investigations in cellular and animal models of neurodegenerative diseases, there are some reports that identified DNA methylation patterns associated with exposure to environmental pollutants in human tissues of individuals affected by neurodegenerative diseases. Regarding exposure to metals, by using a DNA methylation signature specific for lead exposure [117], PD individuals were associated with increased levels of lead-related DNA methylation changes, supporting the hypothesis that chronic and long-term lead exposure may contribute to PD pathogenesis [118]. In a study conducted on aluminum potroom workers, including 43 individuals with mild cognitive impairment, it was found that serum Al concentration negatively correlated with scores on the Mini-Mental State Examination (MMSE), a questionnaire extensively used to measure cognitive impairment, and with peripheral blood global DNA methylation levels [119]. Regarding manganese, Searles Nielsen and collaborators [120] showed that workers exposed to manganese-containing welding fumes who developed parkinsonism had lower mean methylation levels of *NOS2*, a gene involved in inflammation, compared to welders with normal neurological exams. However, in another study, although decreased blood DNA methylation was detected between patients and controls, no differences between PD individuals residing in a city built for the exclusive use of mining companies (unspecified metal type) and PD patients residing in a city having little association with mining was observed [121]. A genome-wide investigation in the peripheral blood of ALS individuals identified DNA methylation marks associated with self-reported exposure to cadmium, mercury, and metallurgy [122]. Particularly, exposure to cadmium was associated with DNA methylation changes in the proximity of *PEX11B* and *ZFR2* genes, involved in peroxisomal metabolism and RNA binding activity, respectively, and to decreased methylation of a CpG site close to the *P2RY6* gene encoding for a receptor activated by extracellular nucleotides. Metallurgy was associated with hypermethylation of a CpG site close to the *PRKG1-AS* gene, encoding a long non-coding RNA. Mercury exposure, like cadmium exposure, was associated with increased methylation of the *PEX11B* gene and hypomethylation of an intergenic CpG site on chromosome 12 [122]. Moreover, a more recent study identified DNA methylation changes in the peripheral blood of ALS patients that were associated with several chemical compounds including various metals such as sodium arsenite, silicon dioxide, and nickel, as well as particulate matter, lifestyle factors such as smoking, and chemical agents used as pesticides such as rotenone and dichlorodiphenyltrichloroethane (DDT) [123].

One of the first studies that suggested a link between exposure to pesticides and neurodegeneration through the action of epigenetic mechanisms dates back to 2009. In that study, the authors searched for global DNA methylation differences in brain tissues from a pair of monozygotic twins discordant for AD [124]. Decreased global DNA methylation levels were detected in the temporal neocortex neuronal nuclei of the AD twin, but not in the cerebellum, a brain region that is not involved in AD pathology. Authors reported that the AD twin was a chemical engineer with extensive pesticide exposure during his life, while the non-demented twin lived in a different work environment, thus suggesting that the prolonged exposure to pesticides could be one of the factors that played a role in the different health outcomes of the twins [124]. The potential involvement of pesticides in AD through DNA methylation alterations has been further suggested by an EWAS investigation in the peripheral blood DNA of 237 individuals with different exposures to pyrethroid pesticides [125]. Several CpG sites associated with pyrethroid exposure were identified, and some of these sites were mapped to loci involved in different diseases, including cancer, diabetes, and AD [125]. Moreover, although to a lesser extent, some differentially methylated genes were associated with other neurological disorders, including PD and ALS [125]. It should be emphasized that this study was not conducted on patients with neurodegenerative diseases, but on subjects from the general population, and it is therefore not possible to have a direct link with the diseases. So, these studies do not allow the provision of clear evidence of an association between pesticide exposure and AD through epigenetic mechanisms, but they suggest that pesticide-induced alteration in DNA methylation could contribute to the neurodegenerative process. More evidence of the role of epigenetic mechanisms underlying the relationship between pesticide exposure and neurodegeneration has been observed in PD patients. An EWAS investigation performed in peripheral blood samples of 580 subjects, including 342 PD patients and 238 controls, with different exposure to organophosphates (OP) insecticides, identified 70 CpG sites associated with the exposure, of which 7 were specific to PD patients [126]. Among the genes specifically associated with PD were *MYH15*, involved in muscle contraction, *MFAP2*, encoding for a component of the elastin-associated microfibrils, and *KIAA0319*, which is involved in neuronal migration during the development of the cerebral neocortex and may play a role in adhesion between migrating neurons. Other PD-specific OP-related CpGs were intergenic or in genes of unknown function [126]. Subsequently, an EWAS investigation in matched peripheral blood and post-mortem brain specimens of PD patients who were plantation workers exposed to organochlorine pesticides identified differentially methylated regions in both tissues that were associated with the time of exposure [127]. Authors observed that altered methylation was associated with genes involved in immune, proinflammatory, and protein clearance pathways. A recent EWAS analysis performed in the peripheral blood of agricultural workers that included PD patients and control subjects showed that exposure to pesticides influenced DNA methylation in PD patients, particularly in females, suggesting an influence of sex in the interaction between pesticides and disease onset [128]. Furthermore, as described above, DNA methylation changes linked to both rotenone and DDT exposure were also observed in blood DNA samples from ALS patients [123].

Regarding air pollution, a genome-wide investigation performed in post-mortem brain tissue recently identified twenty-four CpG sites as mediators of the association between PM_2.5_ exposure and neuropathology markers of AD [129]. It should be underlined that ambient fine particulate matter is a complex mixture of many components (PMCs) that differ in their physicochemical, and toxicological properties and that could exert different epigenetic modifications. For example, Wang and collaborators performed an EWAS analysis in the peripheral blood of 669 men, searching for an association between DNA methylation and PMCs [130]. The authors identified specific probes, regions, and pathways associated with specific PMCs. For example, iron included in PM was associated with methylation levels of 6 probes and six regions, whereas nitrate was associated with 15 probes and three regions, thus suggesting that each component of particulate matter could induce specific DNA methylation signatures [130]. DNA methylation changes linked to PM exposure were also observed in blood DNA samples from ALS patients [123].

Overall, we collected twelve reports aimed at investigating associations among exposure to metals, pesticides, or air pollutants in tissues, DNA methylation changes, and neurodegenerative diseases in human samples (Table 2). The majority of them (n = 6) were performed in the peripheral blood of PD patients, one in post-mortem brain specimens of AD patients, two in the peripheral blood of ALS patients, and one in post-mortem individuals characterized for AD-related neuropathological markers. We also included a study performed in the peripheral blood of healthy subjects exposed to pesticides in whom altered methylation levels of genes associated with AD had been identified [125]. Studies in human subjects affected by the disease could provide information on the causality between the exposure and the health status outcome mediated by epigenetic mechanisms. For example, the studies performed in the peripheral blood of PD patients strongly suggest a role for DNA methylation alterations in the disease promotion following exposure to Pb [118], Mn [120], and pesticides [126,127,128]. However, the results obtained until now still did not allow the establishment of solid epigenetic biomarkers in the disease–exposure relationship. The evidence obtained so far comes from single studies, and it is not currently possible to compare the results of the various studies, given the differences in study designs. Thus, although the results obtained so far are encouraging, further studies are needed to obtain solid epigenetic biomarkers of exposure related to neurodegenerative diseases.

Overall, studies performed until now in cellular and animal models and in human samples of neurodegenerative diseases show that exposure to metals, pesticides, and air pollution is associated with altered DNA methylation levels. However, the molecular mechanisms through which environmental factors could induce alteration in DNA methylation levels have not been investigated in those studies. For certain environmental factors, the potential cascade of events, starting with exposure to pollutants and leading to epigenetic changes, has been outlined. Exposure to metals, pesticides, and air pollution can induce increased ROS production, which can impact DNA methylation through the direct modification of DNA bases. For example, hydroxyl radicals can convert 5mC into 5hmC by abstracting a hydrogen atom from the methyl group [131]. 5hmC can then undergo further modifications (Figure 1) and become cytosine, thus losing the epigenetic information. ROS can also influence DNA methylation by oxidizing guanosine to 8-oxo-2′-deoxyguanosine (8-oxodG). If 8-oxodG is formed in a CpG site context, it can create steric hindrance that prevents the methylation of the adjacent cytosine, inducing DNA hypomethylation [132]. Furthermore, it has been observed that 8-oxoguanine DNA glycosylase-1 (OGG1), which is the enzyme that removes 8-oxodG residues, can promote DNA demethylation by interacting with and TET1 and recruiting it to 8-oxoG lesions [133]. Increased levels of 8-oxodG and a consequent increase in *APP* gene expression through the DNA demethylation of its promoter have been proposed as the mechanism underlying AD induction following developmental exposure to Pb [134]. Such a mechanism, which could be triggered not only by Pb but also by other environmental stressors, is at the basis of the “Latent Early Life-Associated Regulation” (LEARn) model, which hypothesizes that early-life environmental exposures can modify the methylation and expression of specific genes, thereby determining susceptibility to oxidative DNA damage in the aging brain [135]. Moreover, ROS can inhibit the activity of DNMTs by reducing the availability of SAM, thus inducing global DNA hypomethylation [136]. Global DNA demethylation, in addition to triggering the expression of specific genes by hypomethylating their promoters, is associated with chromosomal instability, a characteristic of neurons in neurodegenerative diseases [124,137]. Interference with the activity of DNMTs has also been observed as the direct consequence of the intracellular metabolism of pollutants. For example, in order to be eliminated from the body, arsenic must be metabolized to methylarsonic (MMA) acid and dimethylarsinic acid (DMA) in a reaction catalyzed by DNMTs whose substrate is SAM [138]. Of note, MMA and DMA are the active compounds of a wide range of herbicides, known pollutants associated not only with PD, but also with AD and ALS [139].

Exposure to metals, pesticides, and air pollutants could also impair epigenetic mechanisms through interference with mitochondrial activity [140]. Indeed, mitochondria provide key molecules for the epigenetic machinery, such as ATP, acetyl-CoA, and α-ketoglutarate, so mitochondrial dysfunction may dysregulate the epigenome and gene expression [141]. Furthermore, epigenetic mechanisms within the mitochondria themselves can be perturbed following exposure to environmental pollutants [60]. Of note, only one study performed in rats investigated the potential involvement of mtDNA methylation in environment-induced neurodegeneration [103], and no studies in human subjects suffering from neurodegenerative diseases have been performed until now. Although studies on patients with neurodegeneration are lacking, numerous investigations showed that mtDNA methylation seems to be particularly sensitive to environmental pollutants, particularly to air pollution. The first evidence of such an association was proven by a study that investigated methylation levels of the transfer RNA phenylalanine (*MT-TF*) and *MT-RNR1* genes which increased in the peripheral blood of workers exposed to high levels of PM_1_ [142]. The authors also observed that *MT-RNR1* methylation levels positively correlated with mtDNA copy number. Following this, a study performed in the peripheral blood of 101 welders highly exposed to respirable dust and 127 control subjects identified a decreased methylation level of the D-loop region and a concomitant increase in mitochondrial DNA copy number [143]. Moreover, PM_2.5_ levels induced increased D-loop methylation and decreased mtDNA content in the placenta of women exposed during pregnancy [144]. Exposure to PM_2.5_ was also negatively associated with D-loop methylation levels measured in the peripheral blood of welders [145] and inversely associated with methylation in the *MT-ATP6* and *MT-ATP8* genes in diesel engine manufacturers [146]. Moreover, significant DNA methylation alterations throughout the mitochondrial genome have been recently observed following short exposure to O_3_, CO, and SO_2_ in platelet mtDNA [147]. As reported above (Section 4), there is accumulating evidence of an alteration in the levels of mtDNA methylation in patients with neurodegenerative diseases. Interestingly, the D-loop region of mtDNA, whose methylation levels are particularly sensitive to environmental factors, has also been identified by multiple studies as epigenetically dysregulated in both AD and ALS. So, it could be speculated that environmental factors may influence D-loop methylation, leading to alterations in mitochondrial DNA transcription and replication, ultimately resulting in mitochondrial dysfunction. While the exact impact of environmental factors on mitoepigenetic mechanisms remains unclear, recent observations suggest that mitochondrial DNA methylation could serve as a protective mechanism against oxidative damage [59]. Thus, changes in mitochondrial DNA methylation might represent a defensive response initiated by the mitochondria to react to adverse environmental exposures, such as metals, pesticides, and air pollutants, that are known to induce inflammation and oxidative stress.

## 6. Conclusions and Future Perspectives

This review aimed to collect the available literature suggesting an involvement of altered DNA methylation in neurodegeneration induced by metals, pesticides, and air pollutants. We selected studies performed in animal and cell culture models and in human specimens from major neurodegenerative diseases, namely AD, PD, and ALS. Although the majority of the considered environmental factors have likely a role in the etiology of these disorders, and epigenetic properties have been shown for all of them, studies aiming to directly link DNA methylation changes observed in patients to their exposure are still relatively few and limited. Moreover, the genome targets and disease models used are highly heterogeneous, and humans are simultaneously exposed to different compounds, so it is difficult to draw clear and definitive conclusions about the capability of a certain environmental factor to induce epigenetic changes potentially contributing to neurodegeneration. Therefore, it is currently not possible to perform a quantitative evaluation of the environmental factors that induce neurodegenerative diseases through modifications of DNA methylation levels. Future studies should provide dose–effect information to effectively show how exposure to metals, pesticides, and air pollutants can induce epigenetic alterations that contribute to the etiopathogenesis of neurodegenerative diseases. Moreover, future studies should also implement gene expression investigations to better appreciate the effects of the DNA methylation changes detected. However, although a causal role cannot yet be confirmed, the numerous studies described in this article highlight the epigenetic potential of these compounds, and, in some cases, a direct link between a particular exposure and DNA methylation changes of disease-related genes is emerging, as in the case of early-life exposure to lead. Therefore, further investigation is warranted to better address this issue.

The challenge for the future will be to identify specific epigenetic signatures for the different environmental factors, which could provide biomarkers of exposure, as well as biomarkers of the pathological pathways involved in the induced adverse effects on health. Although we are only at the beginning, the results obtained so far are promising, and the directions to take are outlined. In this regard, Colicino and collaborators [117], developed a blood-based DNA methylation biomarker for lead exposure by using information on skeletal lead content measured with fluorescence spectroscopy and genome-wide methylation analysis in peripheral blood. Although fluorescence spectroscopy is the gold standard technique for measuring bone lead content, it is an expensive tool that cannot be used in large-scale epidemiological studies. If the epigenetic signature developed for lead exposure is confirmed by further studies, this could have a major impact on investigations aimed at quantifying the potential exposure to the metal in the general population.

However, an individual is likely exposed to a myriad of environmental factors during his/her life, and the effects on his/her health are probably the results of the synergistic effects of the different factors. For example, recent meta-analytic research has identified 12 non-genetic high-risk factors for dementia, including low educational attainment in early life, hearing loss, excessive alcohol consumption, hypertension, traumatic brain injury, smoking, obesity in middle age, social isolation, physical inactivity, depression, diabetes, and air pollution, which if prevented could reduce the risk of dementia by around 40% [5]. However, it remains to be clarified how much and in what way each of these factors contributes to the disease. In this manuscript, we focused on specific environmental pollutants, including metals, pesticides, and air pollutants. Nevertheless, other environmental factors known to contribute to neurodegeneration may also exert their neurotoxic effects through alterations in DNA methylation. For instance, cyanobacterial β-N-methylamino-l-alanine (BMAA) has been linked to the development of AD, PD, and ALS [37,148,149], with DNA methylation alterations proposed as a potential underlying mechanism [150]. The use of “omics” technologies will probably help identify the main exposures that an individual has experienced and the biological signatures of such exposures. In this way, the term “exposome”, defined as the total amount of environmental exposure of an individual throughout his life, including lifestyle, diet, environmental factors, and stressors, has been recently coined [151]. By applying exosomes, genomics, epigenomics, transcriptomics, metabolomics, and proteomics together, we could obtain a set of information that can help us to deeply understand the effects of exposure to different neurotoxicants on our cells and tissues, and therefore on our state of health.

Another key point that needs to be clarified is the critical time windows during which the brain is most sensitive to neurotoxicant exposure. The majority of the studies on the DNA methylation effects of metals, pesticides, and air pollutants in neurodegenerative diseases were performed in vitro or in tissues of adult or elderly people exposed to the pollutant during adulthood. However, based on DOHaD theory, already during in utero and first months of life, adverse exposure could leave DNA methylation marks able to reprogram the genome of a baby, predisposing him/her to develop chronic diseases in adult life [152]. Interestingly, the study by Wu and coworkers [67], performed on monkeys exposed to lead as newborns, revealed the existence of a temporal window during early life when the brain is particularly vulnerable to the epigenetic consequences of environmental exposure. This issue needs to be further explored, and longitudinal studies are required to clarify if epigenetic changes induced in early life can be reversed by subsequent interventions.

In conclusion, there is evidence suggesting that metals, pesticides, and air pollutants can induce DNA methylation changes and that exposure to them can contribute to the development of neurodegenerative diseases. A better understanding of the epigenetics underlying the neurotoxic effects of these compounds could provide biomarkers of exposure, as well as new targets for preventive or therapeutic interventions.

## Figures and Tables

**Table 1:** In vitro and in vivo studies on DNA methylation changes induced by metals, pesticides, and air pollution in neurodegeneration.

Experimental Model	Disease Model	Epigenetic Target	Results	Reference
**Metals**				
Cortical brain tissue of monkeys exposed to lead as infants, which later developed AD-like pathology by the age of 23	AD	DNMT1 activity	Infantile exposure to lead induced increased expression of *APP* and *BACE1* with concomitant decreased activity of DNMT1 in aged monkeys (23 years old)	[67]
Cortical brain tissue of monkeys exposed to lead as infants, which later developed AD-like pathology by the age of 23	AD	*Dnmts*, *Mecp2*, histone-modifying proteins	Infantile exposure to lead induced increased expression of neurobiology-related genes and decreased expression of Dnmts, MeCP2, and proteins involved in histone modifications	[99]
Human neuroblastoma (SH-SY5Y) cells exposed to manganese as a model of idiopathic PD	PD	Genome-wide DNA methylation	DNA methylation alterations in genes involved in the onset of PD, including hypermethylation of *PINK1*, *PARK2*, and *TH*	[100]
Substantia nigra samples of mice exposed to manganese as a model of PD	PD	Genome-wide DNA methylation	DNA methylation in the promoter region of 226 genes involved in mitochondrial function, cell cycle, DNA damage response, and ion transportation was found to be regulated (mainly hypermethylated) by manganese treatment	[101]
Rat dopaminergic cells treated with manganese and MPP^+^ as models of PD	PD	Genome-wide DNA methylation	Manganese mainly induced hypermethylation in genes involved in cell differentiation and lipid metabolism. MPP^+^ mainly induced hypermethylation in genes involved in mitochondrial function, autophagy/mitophagy, and WNT signaling	[102]
Hippocampal specimens from rats orally exposed to iron levels that cause memory impairment	Neurodegeneration	Global 5-mC and 5-hmC mtDNA content	Iron exposure during the neonatal period at doses that induced neurodegenerative processes led to decreased 5-mC and 5-hmC levels in mtDNA in adulthood	[103]
**Pesticides**				
Substantia nigra of mice developmentally exposed to dieldrin to model an early stage of PD	PD	Genome-wide DNA methylation	Developmental dieldrin exposure induced altered methylation levels in several genes, including hypermethylation of *Nr4a2* and hypomethylation of *Lmx1b* which are involved in dopaminergic neuron development and maintenance	[105]
Substantia nigra of mice developmentally exposed to dieldrin to model an early stage of PD	PD	Methyl-sequencing of targeted regions	Dieldrin induced DNA methylation changes in pup mice exposed to the pesticide in utero and after birth until 36 weeks of age in a sex-specific manner in loci associated with pathways related to neurodevelopment, dopaminergic neuron differentiation, synaptogenesis, and synaptic plasticity	[108]
Human embryonic kidney cell with a neuronal lineage phenotype treated with rotenone as a model of PD	PD	Genome-wide DNA methylation	Rotenone treatment induced hypomethylation of genes involved in neuronal development and maturation, including *HCN2* and *NEFM*	[109]
Human embryonic kidney cell with a neuronal lineage phenotype treated with rotenone as a model of PD	PD	Genome-wide methylation	Altered methylation in 45 CpG sites (53% hypermethylated) surrounding CTCF binding sites in 7 PD-associated genes, including *BMP4*, *UBOX5*, *GPRIN3*, *FER*, *CNKSR3*, *PARK2*, and *CHCHD2*	[113]
Midbrain of mice exposed to fenpropathrin as a model of PD	PD	Genome-wide DNA methylation	Hypermethylation of the *Ambra1* gene, which in turn was downregulated, led to dopaminergic neuron damage through the Ambra1/Parkin/LC3B-mediated mitophagy pathway	[114]
**Air pollution**				
Hippocampus samples of aged mice exposed to traffic-related air pollution which developed impairment in memory function	AD	*Abca7* and *Pyk2* genes	Increased methylation levels of *Abca7* and decreased methylation of *Pyk2* genes, together with altered mRNA expression levels	[116]

Abbreviations: AD, Alzheimer’s disease; CTCF, Transcriptional repressor CTCF; MPP^+^, 1-methyl-4-phenylpyridinium; PD, Parkinson’s disease.

**Table 2:** Human studies on DNA methylation changes induced by metals, pesticides, and air pollution in neurodegeneration.

Model	Exposure	Disease	Epigenetic Target	Results	Reference
**Metals**					
Peripheral blood of PD (*n* = 1528) and control subjects (*n* = 1169)	Lead	PD	Genome-wide DNA methylation	PD patients had increased DNA methylation-predicted lead levels in tibial bone	[118]
Peripheral blood of 366 Al potroom workers, including 43 MCI	Aluminium	MCI	Global DNA methylation	Increased aluminum serum levels were inversely correlated with global DNA methylation and MMSE	[119]
Peripheral blood of 201 workers, including 49 with parkinsonism	Manganese	PD	*NOS2* gene methylation	Workers with parkinsonism had lower mean methylation levels of *NOS2*, a gene involved in inflammation	[120]
Peripheral blood samples of 45 PD patients and 52 control subjects	Heavy metal mining	PD	Global 5-mC	Decrease in global DNA methylation in PD patients either exposed or not exposed to mining activity. No difference between exposed and non-exposed PD patients	[121]
Peripheral blood samples of 438 ALS cases and 417 controls	Cadmium, mercury, and metallurgy	ALS	Genome-wide DNA methylation	Self-reported cadmium, mercury, and metallurgy exposure was associated with methylation levels of seven CpG sites across the genome in ALS, including hypermethylation of *GNRHR2*, *PEX11B*, *ZFR2*, *LINGO1*, and *PRKG1-AS1* and hypomethylation of *P2RY6* and *KSR2* genes	[122]
Peripheral blood of 61 ALS patients and 61 controls	Various chemicals, metals, pesticides, and air pollutants	ALS	Genome-wide DNA methylation	ALS epigenetic signature associated with exposure to various metals, including sodium arsenite and nickel, air pollutants, including PM, and pesticides, including rotenone	[123]
**Pesticides**					
Brain tissue from a pair of monozygotic twins discordant for AD	Pesticides	AD	Global DNA methylation	Global DNA hypomethylation in the AD twin who worked in contact with pesticides	[124]
Peripheral blood DNA of 237 individuals	Pesticide (pyrethroid)	Control subjects	Genome-wide DNA methylation	Several CpG sites were associated with pyrethroid exposure, some in genes related to AD, PD, and ALS pathology, including the *RTN3* and *TMOD3* genes	[125]
Peripheral blood samples of 342 PD patients and 238 controls	Pesticides (organophosphate insecticides)	PD	Genome-wide DNA methylation	70 CpG sites were associated with pesticide exposure, of which 7 were specific to PD patients, such as the *MYH15*, *MFAP2*, and *KIAA0319* genes	[126]
Matched peripheral blood and post-mortem brain in 20 PD cases	Pesticides	PD	Genome-wide DNA methylation	By comparing individuals exposed more than 10 years and 0 years, 7 and 123 DML in brain and blood DNA, respectively, were identified. DML were mainly associated with genes involved in neurotoxic and neuropathologic pathways	[127]
Peripheral blood of agricultural workers, including 71 early-stage PD cases and 147 control subjects	Pesticides	PD	Genome-wide DNA methylation	Pesticide exposure influenced blood DNA methylation in females at the early stages of PD, in various genes including the *NFATC1* and *DLGAP1* genes	[128]
**Air pollution**					
Prefrontal cortex tissue of 159 donors evaluated for AD-related neuropathological markers	PM_2.5_	AD	Genome-wide DNA methylation	PM_2.5_ exposure induced altered DNA methylation of twenty-four CpG sites that were associated with neuropathology markers of AD. Several CpG sites were located in genes related to neuroinflammation, including *SORBS2*, *PDE11A*, and *GABBR1*	[129]

Abbreviations: ALS, amyotrophic lateral sclerosis; AD, Alzheimer’s disease; DML, differentially methylated loci; MMSE, Mini-Mental State Examination; MPP^+^, 1-methyl-4-phenylpyridinium; PD, Parkinson’s disease; PM_2.5_, particulate matter that measure less than 2.5 micrometers (μm).
